# Effects of Obesity and Thrombophilia on the Risk of Abortion in Women Undergoing *In Vitro* Fertilization

**DOI:** 10.3389/fendo.2020.594867

**Published:** 2020-12-23

**Authors:** Matteo Candeloro, Marcello Di Nisio, Adalisa Ponzano, Gian Mario Tiboni, Nicola Potere, Marco Tana, Anne W. S. Rutjes, Ettore Porreca

**Affiliations:** ^1^ Department of Medical, Oral and Biotechnological Sciences, University G. D’Annunzio, Chieti-Pescara, Italy; ^2^ Department of Medicine and Ageing Sciences, University G. D’Annunzio, Chieti-Pescara, Italy; ^3^ Department of Vascular Medicine, Academic Medical Center, Amsterdam, Netherlands; ^4^ Unit of Gynecology and Obstetrics, “Floraspe Renzetti” Hospital, Lanciano, Italy; ^5^ Unit of Internal Medicine, “SS Annunziata” Hospital, Chieti, Italy; ^6^ Institute of Social and Preventive Medicine (ISPM), University of Bern, Bern, Switzerland

**Keywords:** obesity, thrombophilia, *in vitro* fertilization, abortion, pregnancy

## Abstract

**Introduction:**

Obesity is associated with a higher risk of abortion in women undergoing *in vitro* fertilization (IVF). Whether thrombophilia amplifies this risk is currently unclear. The aim of this study was to evaluate the effects of thrombophilia on the risk of abortion in obese women treated with IVF.

**Methods:**

Patient characteristics, presence of inherited or acquired thrombophilia, and comorbidities were prospectively collected before the procedure in consecutive women undergoing IVF. The primary outcome was the incidence of abortion among women who achieved a clinical pregnancy.

**Results:**

A total of 633 non-obese and 49 obese Caucasian women undergoing IVF were included. 204 (32%) women achieved clinical pregnancy, of whom six had an ectopic pregnancy and 63 experienced an abortion. The incidence of abortion was higher in obese women compared to non-obese women after adjusting for age (64.3% vs. 29.3%, odds ratio [OR] 4.41; 95% CI 1.41 to 13.81). Women with one or more thrombophilia were at increased risk of abortion relative to those without thrombophilia (OR 2.70; 95% CI 1.34 to 5.45), and the risk seemed to be higher with hereditary (OR 5.12; 95% CI 1.77 to 14.8) than acquired thrombophilia (OR 1.92; 95% CI 0.52 to 5.12; p for interaction 0.194). Among obese women, the presence of one or more thrombophilia seemed associated with a substantially increased risk of abortion (unadjusted OR 14.00; 95% CI 0.94 to 207.6).

**Conclusions:**

Obese women undergoing IVF have a high risk of abortion which seems further amplified by the concomitant presence of thrombophilia.

## Introduction

The overall number of assisted reproductive technology (ART) procedures has constantly grown during the last decades, however, the average proportion of successful attempts remains relatively low (1). The reasons behind the high rates of implantation and placentation failure remain largely unknown.

High body mass index (BMI) is one of the most extensively investigated risk factors which may have a negative impact on fertility treatment outcomes (2). Several cohort studies consistently reported an increased risk of abortion in obese women undergoing *in vitro* fertilization (IVF) compared to normal weight women. In a recent meta-analysis evaluating the correlation between high BMI and ART outcomes, women with BMI >30 kg/m^2^ had 20% lower live birth rate and 50% higher miscarriage rate relative to women with normal BMI (2). The low-grade chronic inflammation and hypercoagulability state that are associated with obesity may increase the risk of thrombotic complications and have negative effects on reproduction (3–5). The procoagulant state could result in the thrombotic occlusion of maternal vessels leading to impaired intervillous space perfusion and placentation failure.

In principle, the presence of thrombophilia could amplify the pro-thrombotic tendency of obese women and the risk of IVF failure. Thrombophilia is a heritable or acquired abnormality of blood coagulation that increases the risk of thrombosis. Both inherited and acquired thrombophilia have been associated with recurrent pregnancy loss and pregnancy complications, and preliminary evidence suggested that thrombophilia may increase the risk of IVF failure, although data remain conflicting (6–8).

The aims of this study were to assess IVF outcomes in obese women compared to non-obese women, and evaluate whether the risk of abortion and IVF failure may be increased by the presence of concomitant thrombophilia.

## Materials and Methods

### Study Population

The main characteristics of the study population have been described previously (7, 9). Briefly, consecutive women <40 years who were scheduled for IVF at our University Center of Reproductive Medicine, Ortona General Hospital, Chieti, Italy from March 2015 to July 2017 were eligible. Exclusion criteria were ongoing anticoagulation or indication for anticoagulant treatment, embryo transfer not performed, and ovarian hyper-stimulation syndrome. In addition, women who did not undergo any of the routine screening for thrombophilia were excluded. At the initial study visit before IVF, we collected information on demographics, comorbidities, personal obstetric history, cause of infertility, prior IVF attempts, thrombophilia screening results, and concomitant medications.

Patients underwent controlled ovarian stimulation and follicle growth monitoring. Ovum pick-up was scheduled 36 h after recombinant human chorionic gonadotropin injection. The embryo transfer was performed under ultrasound abdominal guidance about 72–76 h after ovum pick-up. In accordance with national guidelines, a maximum of three embryos were transferred in uterus. The luteal phase was supported with daily intramuscular injections of progesterone 100 mg (Prontogest, IBSA, Italy) from the day of ovum pick-up until the beta human chorionic gonadotropin serum test was performed at 14th day after the embryo transfer. All IVF procedures were performed by intracytoplasmic sperm injections, and each woman was included only once in the study. A biochemical pregnancy was diagnosed in case of detection of beta human chorionic gonadotropin in serum or urine. Clinical pregnancy was defined as the presence of one or more intrauterine sacs, and the clinical pregnancy rate represented the proportion of women with a clinical pregnancy on the total number of patients who underwent embryo transfer. The live birth rate was defined as the number of deliveries resulting in at least one alive baby out of the number of all patients who underwent embryo transfer.

Blood samples were collected in 3.8% trisodium citrate tubes and centrifuged at 4,000 g for 15 min to obtain platelet-poor plasma. The thrombophilia panel that was routinely requested included hereditary thrombophilia such as factor V Leiden, prothrombin G20210A mutation, deficiency of protein C, protein S, or antithrombin, and acquired thrombophilia including lupus anticoagulant, and anti-cardiolipin and anti-beta2 glycoprotein antibodies. Thresholds for positivity are described in our previous reports (7, 9). The study was approved by the local institutional review board, and all women signed a written informed consent before study procedures.

### Outcomes

The main outcome of the study was spontaneous abortion (or miscarriage), defined as intrauterine pregnancy demise confirmed by ultrasound or histology (10). Early abortion was defined as spontaneous pregnancy termination prior to 10 weeks’ gestation (10). The occurrence of abortion was evaluated in all women who achieved a clinical pregnancy, except those with an ectopic pregnancy.

The secondary outcome was IVF failure, which was defined as a failure to conceive a child among all patients who underwent embryo transfer.

### Statistical Analysis

In the descriptive analyses, continuous variables are presented as means (standard deviation), and categorical variables as numbers (percentage). Differences in baseline characteristics between obese women and non-obese women were tested with the Mann-Whitney and Chi-squared test, as appropriate. BMI was calculated in kg/m^2^ from patients’ height and weight, and used to classify women into two categories: non-obese women with BMI <30 kg/m^2^ and obese women with BMI ≥30 kg/m^2^. The association with abortion and IVF failure was first described with unadjusted odds ratios (OR) and corresponding 95% confidence intervals (95% CI). We performed exploratory analysis with multivariable logistic regression using two different models: Model 1 used binary predictor variables for obesity (obese versus non-obese women) or thrombophilia (one or more thrombophilia versus none) adjusting each of them for age as a continuous variable, and Model 2 that included both binary variables, age as a continuous variable, and an interaction term to determine if the effects of thrombophilia differed by obesity status. In secondary analysis, the risks of abortion and IVF failure were evaluated in patients with hereditary versus acquired thrombophilia. Wald test P values were reported for the effects of obesity, thrombophilia and age. Survival curves for abortion were calculated using the Kaplan‐Meier method with patients right censored at the time of delivery. A p-value of 0.05 (two tailed) was considered significant. All analyses were performed with RStudio, Version 1.1.423 – ^©^ 2009-2018 RStudio, Inc.

## Results

Out of 687 women undergoing IVF, five were excluded because BMI was not available (n=3) or patient was lost to follow-up (n=2). A total of 682 women were considered in the final analysis, including 633 (92.8%) non-obese and 49 (7.2%) obese women. In this latter group, 37 (76%) women had mild obesity (BMI between 30 kg/m^2^ and 35 kg/m^2^), 10 (20%) had moderate obesity (BMI between 35 kg/m^2^ and 40 kg/m^2^), and 2 (4%) had severe obesity (BMI above 40 kg/m^2^) (11).

The baseline characteristics of the study population are reported in **Table 1**. Mean age was 34.6 years ( ± 3.26) and mean BMI was 22.9 kg/m^2^ ( ± 4.15). Age, IVF indication, previous pregnancies and IVF cycles, and the number of transferred embryos were similar between obese and non-obese women (**Table 1**).

**Table 1:** Baseline characteristics of study population.

	Overall	Obese women	Non-obese women	p-value
	N = 682	N = 49	N = 633	
Age, years	34.6 (3.26)	34.8 (3.6)	34.6 (3.2)	0.643
BMI, kg/m2				
Smoking				0.266
Current	98 (14.4)	5 (10.2)	93 (14.7)	
Previous	11 (1.6)	2 (4.1)	9 (1.4)	
Hypertension	5 (0.7)	2 (4.1)	3 (0.5)	0.047
Diabetes	1 (0.1)	1 (2.0)	–	0.097
Aspirin	6 (0.9)	–	6 (0.9)	1
IVF indication				0.088
Ovulatory	59 (8.7)	4 (8.2)	55 (8.7)	
Tubaric	150 (22.0)	8 (16.3)	142 (22.4)	
Endometriosis	55 (8.1)	–	55 (8.7)	
Male	196 (28.7)	15 (30.6)	181 (28.6)	
Idiopathic	144 (21.1)	11 (22.4)	133 (21.0)	
Mixed	61 (8.9)	9 (18.4)	52 (8.2)	
Uterine	12 (1.8)	2 (4.1)	10 (1.6)	
Multiple abortion	5 (0.7)	–	5 (0.8)	
Previous IVF cycles				0.208
0	415 (60.9)	34 (69.4)	381 (60.2)	
1	150 (22.0)	12 (24.5)	138 (21.8)	
≥ 2	115 (16.9)	3 (6.1)	112 (17.7)	
Previous pregnancy				
Ectopic	35 (5.1)	2 (4.1)	33 (5.2)	0.905
Spontaneous	60 (8.8)	3 (6.1)	57 (9.0)	0.728
Following ICSI	60 (8.8)	4 (8.2)	56 (8.8)	0.912
Previous abortion				
Before 12 weeks	31 (4.5)	5 (10.2)	26 (4.1)	0.124
After 12 weeks	6 (0.9)	–	6 (0.9)	0.649
Follicle stimulating hormone, IU/mL	8.01 (4.11)	7.60 (3.88)	8.04 (4.13)	0.472
βhCG*, mIU/mL	2818.61 (1401.14)	3095.59 (1390.27)	2797.10 (1,400.78)	0.151
Number of transferred grade A embryos				0.6
0	36 (5.3)	2 (4.1)	34 (5.4)	
1	94 (13.8)	9 (18.4)	85 (13.4)	
≥ 2	552 (80.9)	38 (77.6)	514 (81.2)	
Number of transferred grade B embryos				0.697
0	505 (74.0)	35 (71.4)	470 (74.2)	
1	128 (18.8)	9 (18.4)	119 (18.8)	
≥ 2	49 (7.2)	5 (10.2)	44 (7.0)	
Number of transferred grade C embryos				0.086
0	666 (97.7)	48 (98.0)	618 (97.6)	
1	12 (1.8)	–	12 (1.9)	
≥ 2	2 (0.3)	–	2 (0.3)	
Thrombophilia*	141 (20.7)	18 (36.7)	123 (19.4)	0.007

Values are reported as mean ( ± SD) and numbers (%).

IVF, in vitro fertilization; ICSI, intra-cytoplasmic sperm injection; βhCG, beta human chorionic gonadotropin; *details on thrombophilia type are provided in the **Supplementary Table**.

**Figure 1:**
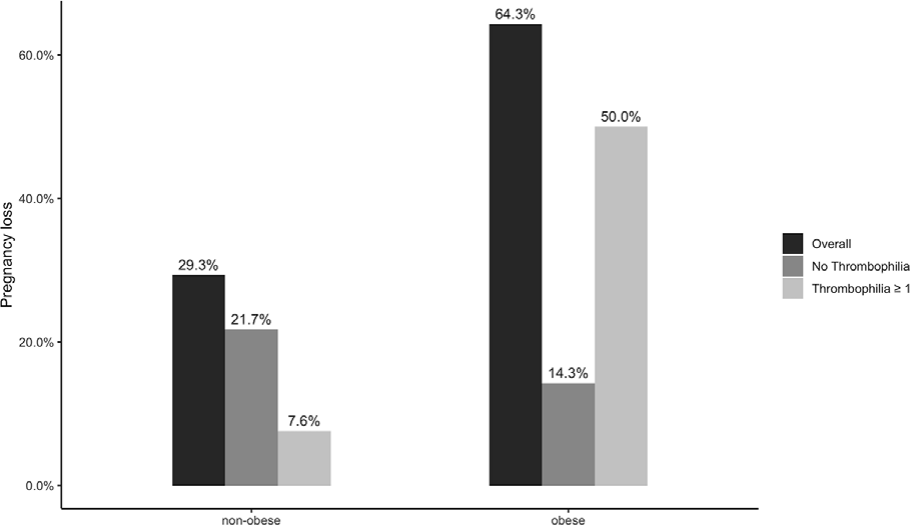
Risk of abortion in non-obese and obese patients according to the presence of thrombophilia.

Overall, 227 (33%) women obtained a biochemical pregnancy, of whom 204 achieved a clinical pregnancy resulting in a clinical pregnancy rate of 29.9%. Eventually, 138 women delivered at least one live baby corresponding to a live birth rate of 20.2%. The proportion of biochemical pregnancy, clinical pregnancy, and live birth rate were comparable between obese and non-obese women (**Table 2**). Six out of 204 women with a clinical pregnancy had an ectopic pregnancy (5 in non-obese women and 1 in obese women, p = 0.913). 63 (32%) of the remaining 198 women experienced an abortion, of whom 54 (29.3%) were non-obese, and nine (64.3%) obese (p = 0.016) (**Figure 1**). Abortion occurred early in eight (57.1%) obese women and in 38 (20.7%) non-obese women.

**Table 2:** *In vitro* fertilization (IVF) outcomes according to the presence of obesity and thrombophilia.

	Overall	Obese women	Non-obese women	p-value*	No thrombophilia	≥ 1 thrombophilia	p-value*
	N = 682	N = 49	N = 633		N = 541	N = 141	
Biochemical pregnancy	227 (33.3)	18 (36.7)	209 (33.0)	0.708	180 (33.3)	47 (33.3)	1
Clinical pregnancy	204 (29.9)	15 (30.6)	189 (29.9)	1	160 (29.6)	44 (31.2)	0.785
Singleton	162 (23.8)	13 (26.5)	149 (23.5)		125 (23.1)	37 (26.2)	
Twin	35 (5.1)	2 (4.1)	33 (5.2)		28 (5.2)	7 (5.0)	
Triplet	7 (1.0)	–	7 (1.1)		7 (1.3)	0 (0.0)	
Ectopic pregnancy	6 (0.9)	1 (2.0)	5 (0.8)	0.913	4 (0.7)	2 (1.4)	
Abortion*	63 (9.2)	9/14 (64.3)	54/184 (29.3)	0.016	42/160 (26.2)	21/44 (47.7)	0.011
Delivery type				0.383			0.055
Vaginal	52 (7.6)	1 (2.0)	51 (8.1)		43 (7.9)	9 (6.4)	
Cesarean	89 (13.0)	5 (10.2)	84 (13.3)		77 (14.2)	12 (8.5)	
Deliveries				0.485			0.33
Singleton	108 (15.8)	5 (10.2)	103 (16.3)		91 (16.8)	17 (12.1)	
Twin	29 (4.3)	1 (2.0)	28 (4.4)		24 (4.4)	5 (3.5)	
Triplet	4 (0.6)	–	4 (0.6)		4 (0.7)	–	
Live birth	141 (71.2)	6 (12.2)	135 (21.3)	0.184	119 (22.0)	22 (15.6)	0.12
IVF failure	544 (79.8)	43 (87.8)	501 (79.1)	0.208	425 (78.6)	119 (84.4)	0.156

Values are reported as numbers (%).

*Abortion was evaluated in women with clinical pregnancy, after excluding women with an ectopic pregnancy. P-values were calculated with Mann-Whitney test for continuous variables and with Chi-squared test for categorical variables.

### Obesity and *In Vitro* Fertilization Outcomes

The risk of abortion was substantially increased in obese women compared to non-obese women (unadjusted OR 4.33; 95% CI 1.39 to 13.53), with an association that remained significant after adjusting for age (OR 4.41; 95% CI 1.41 to 13.81; p = 0.011). The effect of age was negligible (OR 1.03; 95% CI 0.93 to 1.13; p = 0.556). 54.5% of women with mild obesity had an abortion compared to 100% of women with moderate obesity, whereas none of the women with severe obesity achieved a clinical pregnancy. A total of 544 (79.8%) patients had IVF failure, which appeared to be higher in obese than non-obese women, although the association was not statistically significant (unadjusted OR 1.89; 95% CI 0.79 to 4.53). This risk remained similar after adjusting for age (OR 1.88; 95% CI 1.88 0.78 to 4.51; p = 0.16). There was a trend for a small effect of age on IVF failure with 5% increase in risk for each year of age (OR 1.05; 95% CI 1.10 to 1.11; p = 0.074).

### Thrombophilia and *In Vitro* Fertilization Outcomes

Overall, 141 (20.7%) women had at least one thrombophilia consisting in one thrombophilic defect in 121 (17.7%) women, two thrombophilic defects in 17 (2.5%), and three or more thrombophilic defects in three (0.4%) women.

The prevalence of individual thrombophilia ranged from 0.1% to 4.5% with the least represented thrombophilia being factor V Leiden homozygosity which was found in only 1 (0.1%) patient (**Supplementary Table**). Eighteen (36.7%) obese women had one or more thrombophilia compared to 123 (19.4%) non-obese women (p = 0.007). The frequency of individual thrombophilia in the overall study population, obese and non-obese patients is provided in the **Supplementary Table**. In univariable analysis, thrombophilia was associated with an increased risk of abortion (OR 2.71; 95% CI 1.35 to 5.47), and a not statistically higher risk of IVF failure (OR 1.48; 95% CI 0.90 to 2.43). These associations remained similar after adjusting for age (OR for abortion 2.70; 95% CI 1.34 to 5.45; p = 0.005, and OR for IVF failure 1.47; 95% CI 0.89 to 2.43; p = 0.119). The effect of age was estimated with an OR of 1.05 (95% CI 1.00 to 1.11; p = 0.074) for abortion and OR 1.05 (95% CI 1.00 to 1.11; p = 0.073) for IVF failure.

Compared to patients without thrombophilia, women with hereditary thrombophilia had a fivefold higher risk of abortion after adjusting for age (OR 5.12; 95% CI 1.77 to 14.8; p = 0.003). The risk seemed increased also in women with acquired thrombophilia, but the difference did not reach statistical significance (OR 1.92; 95% CI 0.52 to 5.12; p = 0.194).

### Obesity and Concomitant Thrombophilia

Obese women with one or more thrombophilia seemed to have a substantially higher risk of abortion compared to obese women without thrombophilia (unadjusted OR 14.00; 95% CI 0.94 to 207.6), non-obese patients with (unadjusted OR 10.00; 95% CI 1.10 to 90.59) or without thrombophilia (unadjusted OR 19.25; 95% CI 2.30 to 161.39).

**Figure 2:**
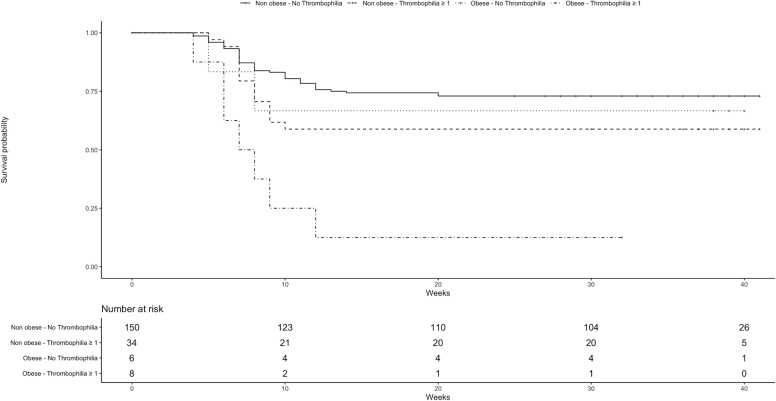
Kaplan-Meyer curves for abortion.

As shown in **Figure 2**, abortion occurred during the first 10 weeks of gestation in 75.0% of obese women with one or more thrombophilia, in 33.3% of obese women without thrombophilia, and in 20.7% of non-obese women.

Model 2 suggested a higher risk of abortion in the presence of obesity and thrombophilia, although the interaction term was not statistically significant (OR 7.46; 95% CI 0.45 to 125.91).

Obese patients with one or more thrombophilia seemed to have an increased risk of IVF failure compared to obese patients without thrombophilia (unadjusted OR 3.27; 95% CI 0.35 to 30.47), non-obese patients with one or more thrombophilia (unadjusted OR 3.50; 95% CI 0.44 to 27.76), and non-obese patients without thrombophilia (unadjusted OR 4.73; 95% CI 0.62 to 35.93), although none of these effects reached statistical significance. The interaction term of obesity and thrombophilia in Model 2 was estimated to be 2.81, but the confidence interval was wide including the value of no effect (95% CI 0.28 to 28.06).

## Discussion

The results of the present study showed that obesity and thrombophilia are both associated with a high risk of abortion in women undergoing IVF. The risk seemed to be substantially increased by the concomitant presence of thrombophilia and obesity, and seemed to be particularly high during the first 10 weeks of gestation.

The association between obesity and risk of abortion is consistent with previous observations and the findings of a recent meta-analysis on the correlation between BMI and ART outcomes, which showed that women with BMI ≥ 30 kg/m^2^ had 50% higher risk of miscarriage compared to those with normal BMI. (2) Other studies did not observe a significant relationship between obesity and abortion or reported a lower risk in patients with elevated BMI (12, 13). The discrepant findings may be attributable to differences in study population, procedures, outcome definition, and type of statistical analyses (12, 13). In an earlier retrospective cohort by Zhang and colleagues, the abortion rate was not statistically different between obese, overweight, and normal weight women (11.1%, 13.6%, 8.4% respectively; p > 0.05) (13). Interestingly, only 1% of 2,628 patients included in that study were obese, which is significantly lower compared to the proportion of obese women evaluated in the current and previous cohorts (2).

Our findings extend earlier observations suggesting that the risk of abortion is amplified in obese women who have concomitant thrombophilia. Obesity and thrombophilia may have synergistic effects and activate blood coagulation beyond the physiological state of hypercoagulability characteristic of pregnancy, potentially increasing the risk of placental vessels thrombosis, pregnancy loss, and pregnancy complications (6–8, 14). Our analysis is limited by the relative low number of obese women included, which resulted in wide confidence intervals around the effects of thrombophilia in these patients. If confirmed, our data may have implications for the counselling of obese women seeking IVF treatment. Thrombophilia testing could be considered in these patients to inform them about the effects of increased BMI and concomitant thrombophilia on IVF outcomes and increased risk of early abortion. While speculative, these findings support the hypothesis that antithrombotic treatment may improve IVF outcomes in obese patients with thrombophilia. Preliminary data suggested that the administration of low-molecular-weight heparin is associated with higher clinical pregnancy and live birth rates in women undergoing IVF or intracytoplasmic sperm injection. (14). The use of heparin would be an attractive treatment option in obese women undergoing IVF, who seem to derive less benefit from weight loss compared to obese women conceiving spontaneously (15). The efficacy and safety of antithrombotic therapy has not been evaluated specifically in obese women undergoing IVF, and adequately powered randomized studies are warranted before considering heparin treatment in these patients.

Our study has some limitations that need to be discussed. The number of obese women was relatively low reducing the overall power of the analysis and hampering the model fit in Model 2, thus all estimations should be interpreted cautiously. The low number of obese women precluded the evaluation of the relationship between individual thrombophilia and IVF outcomes. About half of patients lacked evaluation of all thrombophilia, possibly affecting the strength of the associations. Therefore, the apparent higher risk of abortion in women with hereditary thrombophilia may be a spurious effect and requires additional investigation. The incomplete assessment was most often related to the patient’s decision to proceed to IVF avoiding further delays and costs associated with laboratory testing, which also highlights possible challenges for implementing thrombophilia testing in clinical practice.

In conclusion, obesity and thrombophilia were both independently associated with a significantly higher risk of abortion in women undergoing IVF. The presence of concomitant thrombophilia and obesity seemed to amplify this risk substantially. If confirmed, these findings may be useful for risk stratification, patient counselling, improvement of prevention strategies, as well as for designing future interventional studies.

## Data Availability Statement

The datasets presented in this article are available upon request. Requests to access the datasets should be directed to MDN, mdinisio@unich.it.

## Ethics Statement

The studies involving human participants were reviewed and approved by the ethic committee of Chieti and Pescara. The patients/participants provided their written informed consent to participate in this study.

## Author Contributions

MC and MDN conceptualized the study, performed the formal analysis, and wrote the original draft. AR performed the formal analysis, and wrote, reviewed, and edited the manuscript. AP reviewed and edited the manuscript, and performed the data curation. NP and MT reviewed and edited the manuscript. GT and EP wrote, reviewed, and edited the manuscript, supervised the study, and was in charge of the project administration. All authors contributed to the article and approved the submitted version.

## Conflict of Interest

The authors declare that the research was conducted in the absence of any commercial or financial relationships that could be construed as a potential conflict of interest.
