# Supplementary material for: Effects of Obesity and Thrombophilia on the Risk of Abortion in Women Undergoing In Vitro Fertilization
Source: Front Endocrinol (Lausanne). 2020 Dec 23;11:594867. doi: 10.3389/fendo.2020.594867 (PMC7786836; doi:10.3389/fendo.2020.594867)
Supplement: Supplementary file 1 [file Table_1.pdf]

Supplementary Table. Distribution of thrombophilia in obese and non-obese patients

	**Overall**	**Obese women**	**Non-obese women**	**p-value**
	N =682	N = 49	N =633	
No thrombophilia	238 (34.9)	21 (42.9)	217 (34.3)	0.29
Protein S deficiency	15 (2.2)	1 (2.0)	14 (2.2)	0.528
Protein C deficiency	8 (1.2)	-	8 (1.3)	0.513
Prothrombin G20210A mutation	21 (3.1)	2 (4.1)	19 (3.0)	0.734
Factor V Leiden mutation				0.64
Heterozygosity	31 (4.5)	4 (8.2)	27 (4.3)	
Homozygosity	1 (0.1)	-	1 (0.2)	
Antithrombin III deficiency	4 (0.6)	1 (2.0)	3 (0.5)	0.225
Activated protein C resistance	21 (3.1)	3 (6.1)	18 (2.8)	0.257
Anticardiolipin antibodies	31 (4.5)	3 (6.1)	28 (4.4)	0.822
Lupus anticoagulant	20 (2.9)	6 (12.2)	14 (2.2)	<0.001
Anti-beta 2-glycoprotein antibodies	12 (1.8)	1 (2.0)	11 (1.7)	0.106
